# Antidiabetic Effect of *Sida cordata* in Alloxan Induced Diabetic Rats

**DOI:** 10.1155/2014/671294

**Published:** 2014-07-09

**Authors:** Naseer Ali Shah, Muhammad Rashid Khan

**Affiliations:** Department of Biochemistry, Faculty of Biological Sciences, Quaid-i-Azam University, Islamabad 45320, Pakistan

## Abstract

Medicinal plants are efficient ameliorator of oxidative stress associated with diabetes mellitus. In this study, ethyl acetate fraction (SCEE) of *Sida cordata* was investigated for scientific validation of its folk use in diabetes. Antidiabetic effect of SCEE was confirmed by antihyperglycemic activity in normal glucose loaded and diabetic glucose loaded animals as well as normal off feed animals. Confirmation of antidiabetic activity and toxicity ameliorative role of *S. cordata* was investigated in a chronic multiple dose treatment study of fifteen days. A single dose of alloxan (120 mg/kg) produced a decrease in insulin level, hyperglycemia, elevated total lipids, triglycerides, and cholesterol and decreased the high-density lipoproteins. Concurrent with these changes, there was an increase in the concentration of lipid peroxidation (TBARS), H_2_O_2_, and nitrite in pancreas, liver, and testis. This oxidative stress was related to a decrease in glutathione content (GSH) and antioxidant enzymes. Administration of SCEE for 15 days after diabetes induction ameliorated hyperglycemia, restored lipid profile, blunted the increase in TBARS, H_2_O_2_, and nitrite content, and stimulated the GSH production in the organs of alloxan-treated rats. We suggested that SCEE could be used as antidiabetic component in case of diabetes mellitus. This may be related to its antioxidative properties.

## 1. Introduction

Diabetes mellitus is a metabolic disease with deficiency of secretion or action of endogenous insulin, features of hyperglycemia, and no definite cause [1, 2]. Diabetes mellitus is a multifactorial illness with imperfection in reactive oxygen species (ROS) scavenging enzymes [3], lipoprotein abnormalities [4], hyperglycemia [5], high basal metabolic rate [6], and high oxidative stress induced damage [7].

Diabetes in rodents is induced by injecting alloxan or streptozotocin which induce diabetes while producing ROS leading to demolition of pancreas *β*-cells [8, 9]. The prime cause of a number of long term complications of diabetes is chronic hyperglycemia. Protein glycation, the most important source of free radicals, is leaded by hyperglycemia. According to the amount of evidence, it is now known that important contribution to the progression and complications of diabetes is done by free radicals. Changes in metabolism, nerve, kidney, foot ulceration, and vascular tissue comprise these complications [10]. Chronic elevated glucose levels cause diabetic complications in both types 1 and 2 diabetes. ROS produced by protein glycation and glucose oxidation mediates the pathogenic effects of high glucose. ROS can directly impose molecular damage as well as cellular damage by activating many cellular stress-sensitive pathways, which direct to late complication of diabetes. Moreover, *β*-cell dysfunction and insulin resistance also show links to the same pathways. ROS and hyperglycemia can activate JNK/SAPK, NF-*κ*B, p38 MAPK, and hexosamine pathways which are stress-sensitive signaling pathways. These pathways take part in the pathogenesis of diabetes [11].

Many plant secondary metabolites have shown antioxidant potential and have shown ameliorative effect on oxidative stress induced damage in diabetes [5].* Sida cordata* (Burm. f.) synonym,* Sida veronicaefolia* Lam, member of the Malvaceae family [12], is locally known as Farid buti, Rajbala, Bhumibala, and Shaktibala in India and Simak in Pakistan. It is cosmopolitan in India, Pakistan, and other tropical countries and is extensively used for therapeutic purposes in the codified Indian systems of medicine, namely, Siddha and Ayurveda. Its roots are used as diuretic, astringent, stomachic, febrifuge, and demulcent and seeds are applied as laxative, aphrodisiac, and demulcent, recommended in cystitis, colic, gonorrhea, tenseness, and piles [13]. The drug is useful in neurological disorders such as hemiplegia, facial paralysis, sciatica, general debility, headache, ophthalmia, dysuria, leucorrhoea, tuberculosis, diabetes, fever, rheumatism, and uterine disorders [14, 15]. Shah et al. [16] reported its phytochemical, cytotoxic, and* in vitro* antileishmanial activity. Mistry et al. [15] and Shah et al. [17] reported* S. cordata* antioxidant potential as hepatoprotective against CCl_4_ induced toxicity in rat. Shah et al. [17] described ethyl acetate fraction as strong antioxidant component. The present study was undertaken to systematically evaluate the ethyl acetate fraction against alloxan induced diabetes and role in reducing the toxic effects of diabetes on vital organs.

## 2. Materials and Methods

### 2.1. Chemicals

Reduced glutathione (GSH), glutathione reductase, *γ* *-*glutamyl p-nitroanilide, bovine serum albumin (BSA), 1,2-dithio-bis-nitrobenzoic acid (DTNB), 1-chloro-2,4-dinitrobenzene (CDNB), reduced nicotinamide adenine dinucleotide phosphate (NADPH), flavin adenine dinucleotide (FAD), glucose-6-phosphate, 2,6-dichlorophenolindophenol, thiobarbituric acid (TBA), picric acid, sodium tungstate, sodium hydroxide, trichloroacetic acid (TCA) and alloxan, and glibenclamide were purchased from Sigma Chemicals Co., USA. Testosterone kit was purchased from Med Lab Services, Rawalpindi, Pakistan.

### 2.2. Plant Collection

The* S. cordata* whole plant was collected in the month of December 2011 from the campus of Quaid-i-Azam University, Islamabad, Pakistan, and recognized by their local name and then confirmed by Dr. Muhammad Zafar, Curator, Herbarium, Quaid-i-Azam University, Islamabad. Voucher specimen with accession number 27824 was deposited at the Herbarium, Quaid-i-Azam University, Islamabad.

### 2.3. Extraction and Fractionation

After collection, plant sample was shade dried till the complete removal of moisture and was made to mesh sized powder by using plant grinder. Powder (5 kg) was soaked in crude methanol (10 L) for 72 h and was filtered by using Whatman No. 1 filter. Solvent was evaporated on a rotary evaporator at 40°C under reduced pressure.

To sort the compounds in the crude methanol extract with increasing polarity, crude methanol extract (6 gram) was suspended in distilled water (250 mL) and passed to liquid-liquid partition by using solvents in order of* n*-hexane, chloroform, ethyl acetate, and* n*-butanol. The residue left behind was termed as aqueous fraction. Fractions were further dried under reduced pressure and stored at 4°C for phytochemical and pharmacological evaluation.

### 2.4. Antidiabetic Activity

Screening of ethyl acetate fraction “SCEE” for antidiabetic activity was performed on rats by following methodology of Jarald et al. [18] with slight modifications.

#### 2.4.1. Glucose Tolerance Test in Normal Animals

Male Sprague Dawley rats (180–200 g) of seven weeks old were used as animal model in this study. They were maintained in cages at room temperature of 25 ± 3°C with a 12 h light/dark cycle and free access to water and feed. The study protocol was approved (number 0244) by the ethical committee of Quaid-i-Azam University, Islamabad, Pakistan, for laboratory animal care and experimentation.

Normal rats were randomly distributed into three groups having five rats each. The normal control group was treated only with vehicle (1 mL, 5% DMSO). The other two groups were treated with SCEE (150 and 300 mg/kg b.w.). The animals received their respective doses orally by feeding tube number 7. The glucose readings before treatment were termed 0 min readings and after treatment three readings were observed at 60, 120, and 180 min by puncturing tail tip with syringe needle. The blood glucose concentration was measured in unit of mg/dL by using glucometer.

#### 2.4.2. Glucose Tolerance Test in Diabetic Animals

The procedure of glucose tolerance test in normal animals was followed in diabetic animals with the addition of the group of diabetic control and reference drug (glibenclamide) treated group. Glibenclamide at 5 mg/kg b.w. was used as a reference drug [19]. Animals were selected, weighed, and then marked for individual identification. The rats were injected with alloxan monohydrate in saline (0.9% NaCl) at a dose of 120 mg/kg b.w. intraperitoneally to induce diabetes in 8 h fasted [19] male Sprague Dawley rats weighing 180–200 g. After one hour of alloxan administration, the animals were given feed* ad libitum*. A 5% dextrose solution (10 g) was given in feeding bottle for a day to overcome the early hypoglycemic phase. After 72 h, animals with blood glucose levels higher than 220 mg/dL were considered diabetic and were included in the study. Blood samples were collected from the tail vein priorly at 0 min and 60, 120, and 180 min after glucose administration. Blood glucose level was estimated using glucometer.

#### 2.4.3. Hypoglycemic Activity

*Sida cordata* fraction “SCEE” was evaluated for hypoglycemic activity test in normal 8 h fasting animals with free access to water. The glucose readings were recorded at 0 min (preglucose treatment) and at 60, 120, and 180 min after glucose treatment.

#### 2.4.4. Chronic Multiple Study

Alloxan induced diabetic model was selected to confirm the utility of the SCEE in chronic multiple dose experiment in the diabetic rats. Doses of 150 and 300 mg/kg b.w. were selected for SCEE treatment, following our previous study [17].

Animals were divided into five groups. Four groups were comprised of diabetic animals and one group of normal animals. Five rats were included in each group. Diabetes induction and animals' selection were followed as described in* Glucose tolerance test in diabetic animals.*

Group  1: normal control animal received 1 mL of the vehicle (1 mL, 5% DMSO).
Group  2: diabetic animal received reference drug glibenclamide (5 mg/kg b.w.) orally.
Group  3: diabetic control received only vehicle 1 mL orally.
Group  4: diabetic animal received SCEE (150 mg/kg b.w.) orally.
Group  5: diabetic animal received SCEE (300 mg/kg b.w.) orally.

Animals of different groups were given treatment according to their respective group for 15 days. After 15 days, fasting blood glucose concentration was measured and animals were dissected. Blood and organs, that is, pancreas, liver, and testes, were collected. Half of the organs were processed for histological examination while the remaining half were preserved for tissue antioxidant enzymes assays.

*(1) Analysis of Blood Parameters*. Quantitative determination of insulin and glycosylated haemoglobin in rat serum was performed by means of an enzyme-linked immunosorbent assay (ELISA) kit according to the protocol provided.

Serum alkaline phosphatase (ALP), alanine transaminase (ALT), aspartate transaminase (AST), lactate dehydrogenase (LDH), bilirubin, and *γ*-glutamyltransferase (*γ*–GT) were estimated by using standard AMP diagnostic kits (Stattogger Strasse 31b 8045 Graz, Austria) [17].

*(2) Assessment of Tissue Antioxidant Enzymes*. Tissues were homogenized in 10 volume of 100 mM KH_2_PO_4_ buffer containing 1 mM EDTA (pH 7.4) and centrifuged at 12,000 g for 30 min at 4°C. The supernatant was collected and used for enzymatic studies. 

*(a) Catalase Assay (CAT)*. CAT activity was determined by the method of Khan et al. [20] with some modifications. The reaction solution of CAT activity contained 2.5 mL of 50 mM phosphate buffer (pH 5.0), 0.4 mL of 5.9 mM H_2_O_2_, and 0.1 mL tissue homogenate. Changes in absorbance of the reaction solution at 240 nm were determined after one min. One unit of CAT activity was defined as an absorbance change of 0.01 as units/min. 

*(b) Peroxidase (POD) Activity*. Khan et al.'s [21] method was used to determine POD activity spectrophotometrically with minor modifications. An amount of 25 *μ*L of tissue homogenate was added to mixture of 25 *μ*L of 20 mM guaiacol, 75 *μ*L of 40 mM H_2_O_2_, and 625 *μ*L of 50 mM potassium phosphate buffer (pH 5.0). At 470 nm absorbance change was measured. Change in absorbance of 0.01 as units/min defines one unit POD activity. 

*(c) Superoxide Dismutase Assay (SOD)*. SOD activity of tissues was estimated by the method of Shah et al. [17]. Reaction mixture of this method contained 0.1 mL of phenazine methosulphate (186 *μ*M), 1.2 mL of sodium pyrophosphate buffer (0.052 mM; pH 7.0), and 0.3 mL of tissue homogenate. Enzyme reaction was initiated by adding 0.2 mL of NADH (780 *μ*M) and stopped after 1 min by adding 1 mL of glacial acetic acid. Amount of chromogen formed was measured by recording color intensity at 560 nm. Results were expressed in units/mg protein. 

*(d) Glutathione-S-transferase Assay (GST)*. The reaction mixture of glutathione-S-transferase activity consisted of 1.475 mL phosphate buffer (0.1 M, pH 6.5), 0.2 mL reduced glutathione (1 mM), 0.025 mL (CDNB; 1 mM), and 0.3 mL of tissue homogenate in a total volume of 2 mL. The changes in the absorbance were recorded at 340 nm and enzymes activity was calculated as nM CDNB conjugate formed/min/mg protein using a molar extinction coefficient of 9.6 × 10^3^ M^−1 ^cm^−1^ [22]. 

*(e) Glutathione Reductase Assay (GSR)*. Glutathione reductase activity was determined with the protocol of Ahmad et al. [23]. The reaction mixture consisted of 1.65 mL phosphate buffer (0.1 M; pH 7.6), 0.1 mL EDTA (0.5 mM), 0.05 mL oxidized glutathione (1 mM), 0.1 mM NADPH (0.1 mM), and 0.1 mL of homogenate in a total volume of 2 mL. Enzyme activity was quantitated at 25°C by measuring disappearance of NADPH at 340 nm and was calculated as a nM NADPH oxidized/min/mg protein using molar extinction coefficient of 6.22 × 10^3^ . 

*(f) Glutathione Peroxidase Assay (GP*_*X*_). Glutathione peroxidase activity was assayed by the method of Shah et al. [17]. The reaction mixture consisted of 1.49 mL phosphate buffer (0.1 M; pH 7.4), 0.1 mL EDTA (1 mM), 0.1 mL sodium azide (1 mM), 0.05 mL glutathione reductase (1 IU/mL), 0.05 mL GSH (1 mM), 0.1 mL NADPH (0.2 mM), 0.01 mL H_2_O_2_ (0.25 mM), and 0.1 mL of homogenate in a total volume of 2 mL. The disappearance of NADPH at 340 nm was recorded at 25°C. Enzyme activity was calculated as nM NADPH oxidized/min/mg protein using molar extinction coefficient of 6.22 × 10^3^ M^−1^cm^−1^.

*(g) Reduced Glutathione Assay (GSH)*. 1.0 mL homogenate of sample was precipitated with 1.0 mL of (4%) sulfosalicylic acid. The samples were kept at 4°C for 1 hour and then centrifuged at 1200 ×g for 20 min at 4°C. The total volume of 3 mL assay mixture contained 0.1 mL filtered aliquot, 2.7 mL phosphate buffer (0.1 M; pH 7.4), and 0.2 mL DTNB (100 mM). The yellow color developed was read immediately at 412 nm on a Smart Spec TM plus Spectrophotometer. It was expressed as *μ*mol GSH/g tissue [17]. 

*(3) Estimation of Lipid Peroxidation (TBARS)*. The assay for lipid peroxidation was carried out with modified method of Khan et al. [24]. The reaction mixture in a total volume of 1 mL contained 0.8 mL phosphate buffer (0.1 M; pH 7.4) and 0.2 mL homogenate sample. The reaction mixture was incubated at 37°C in a shaking water bath for 1 hour. The reaction was stopped by addition of 1 mL 10% trichloroacetic acid following addition of 1 mL 0.67% thiobarbituric acid. All the tubes were placed in a boiling water bath for 20 min and then shifted to crushed ice-bath before centrifuging at 2500 ×g for 10 min. The amount of malondialdehyde formed in each of the samples was assessed by measuring optical density of the supernatant at 535 nm using spectrophotometer against a reagent blank. The results were expressed as nmol of TBARS/min/mg tissue protein.

*(4) Nitrite/Nitrate Assay*. Nitrite/nitrate was assayed colorimetrically in tissue homogenate by using methodology of Berkels et al. [25]. Promega's griess reagent system is based on the chemical reaction between sulfanilamide and N-1-naphthylethylenediamine dihydrochloride under acidic condition (phosphoric acid) to give bright reddish-purple colored azo-compound which can be measured at 540 nm spectrophotometrically. Using standard curve of sodium nitrite, nitrite concentration in tissue samples was calculated.

*(5) H*_*2*_ *O*_*2*_ *Assay*. The methodology of Pick and Keisari [26] was adopted to determine the H_2_O_2_-mediated horseradish peroxidase-dependent oxidation of phenol red. An aliquot of 100 *μ*L of tissue homogenate was added to 100 *μ*L of 0.28 nM phenol red, 250 *μ*L of 5.5 nM dextrose, 8 units of horse radish peroxidase, and 500 *μ*L of 0.05 M phosphate buffer (pH7.0) and incubated at room temperature for 1 hour. Reaction was stopped by the addition of 100 *μ*L of 10 N NaOH and then tubes were centrifuged for 10 minutes at 800 ×g. Supernatant was collected and absorbance was measured at 610 nm using reagent as blank. The quantity of H_2_O_2_ produced was expressed as nM H_2_O_2_/min/mg tissue based on the standard curve of H_2_O_2_ oxidized phenol red.

*(6) Tissue Protein Estimation*. Total amount of soluble protein in tissue homogenate was determined by method of Lowry et al. [27]. To the tissue homogenate 300 *μ*L of 0.1 M potassium phosphate buffer (pH 7.0) was added in order to dilute the tissue sample. To this mixture 1 mL of alkaline copper solution was added and kept at room temperature. After 10 minutes of incubation, 100 *μ*L of Folin-Ciocalteu phenol reagent was added. Reaction tubes containing test mixtures were then vortexed and again incubated at 37°C for 30 minutes. At 650 nm optical density was measured spectrophotometrically. Total soluble proteins of tissue samples were then determined using standard curve of bovine serum albumin.

*(7) Histopathological Determination*. For microscopic evaluation, tissues were fixed in a fixative (absolute ethanol 60%, formaldehyde 30%, and glacial acetic acid 10%) and embedded in paraffin, sectioned at 4 *μ*m, and subsequently stained with hematoxylin/eosin. Sections were studied under light microscope (DIALUX 20 EB) at 10 and 40 magnifications. Slides of all the treated groups were studied and photographed. A minimum 12 fields of each section were studied and approved by pathologist without saying its treatment nature.

### 2.5. Statistical Analysis

Glucose profiles between different groups and timings were analyzed by GraphPad Prism at probability level of 0.05%. However, the parameters of chronic multiple dose studies were statistically analyzed by using Statistix 8.1: computer software used to determine one-way analysis of variance measured at 0.05% significance level of probability among treatments.

## 3. Results

### 3.1. Antihyperglycemic Activity in Glucose-Loaded Normal Animals

In only glucose-loaded normal animals, increase in blood glucose level was observed to increase till 60 min and decreasing trend was observed at 120 and 180 min. SCEE illustrated significant alteration in glucose level after glucose load at 60, 120, and 180 min in comparison to that of the control group. SCEE demonstrated dose dependent alterations and high dose treated group showed very low glucose concentration when compared to the pretreated blood glucose level (0 min) (Table 1).

### 3.2. Antihyperglycemic Activity against Glucose Load in Diabetic Animals

SCEE showed marked changes (*P* < 0.05) in glucose level in diabetic animals against glucose load in comparison to that of the diabetic control at a time period of 60, 120, and 180 min after administration of glucose. Dose dependent effects were also observed for SCEE. Glucose level continued to increase till 120 min after glucose administration in SCEE and diabetic control groups. At 180 min after glucose administration glucose level was observed below the level at 0 min (preglucose administration) in SCEE at high dose, showing antihyperglycemic effect in diabetic glucose-loaded animals (Table 2).

### 3.3. Hypoglycemic Activity in Fasting Animals

Hypoglycemic activity observed is displayed in Table 3. SCEE demonstrated hypoglycemic activity after 60 min of oral administration of SCEE when compared to that of the normal animal group. The hypoglycemic activity of SCEE was found statistically low in comparison to the standard drug glibenclamide.

### 3.4. Antidiabetic Activity of  SCEE Fraction in Alloxan Induced Diabetic Animals

SCEE antidiabetic activity was further evaluated by chronic experiment of 15 days in alloxan induced diabetic animals.

#### 3.4.1. Effect of SCEE on Insulin and Glucose Level

Insulin and glucose levels of blood plasma are the primary markers of diabetes. Alloxan treatment in diabetic group induced pathological lesion in islets of Langerhans of pancreas and resulted in significantly low insulin secretion from beta cell in comparison to that of the normal control group. SCEE significantly elevated insulin plasma level in a dose dependent manner but it was significantly low in comparison to that of normal and standard glibenclamide treated groups.

The glucose level in diabetic rats was markedly elevated in diabetic group animals in comparison to that of the normal groups. SCEE dose dependently diminished elevated plasma level of glucose and no difference was observed in comparison to control group.

#### 3.4.2. Effect of SCEE on Haemoglobin and Glycosylated Haemoglobin

Haemoglobin and glycosylated haemoglobin are associated with diabetes and especially glycosylated haemoglobin is used as a marker for diabetes. Haemoglobin and glycosylated haemoglobin plasma levels in different groups are given in Table 4. Diabetes caused significant reduction in the quantity of haemoglobin. SCEE oral administration significantly recovered its level in a dose dependent passion equal to glibenclamide treated group but it was significantly lower than that of normal control group. Glycosylated haemoglobin quantity in blood plasma of diabetic animal control group was found to be markedly (*P* < 0.05) elevated. SCEE treatment to diabetic animals significantly reversed glycosylated haemoglobin that was significantly different from that of the normal control group.

#### 3.4.3. Effect on Blood Lipid Profile

There was a significant elevated level of triglycerides in the diabetic animal group compared to that of the normal control animals (Table 5). Triglyceride level exhibited a significant reduction in a dose dependent pattern after administration of* S. cordata* fraction “SCEE.” At the high dose of 300 mg/kg b.w., effect of SCEE on the triglyceride was comparable with glibenclamide treated group (*P* > 0.05). Similarly, total cholesterol level in diabetic animal group was significantly elevated in comparison to that of the normal control group. SCEE treatment significantly reversed the elevated level with nonsignificant difference at a high dose in comparison to the control group. High density lipoprotein is considered good cholesterol type and was significantly reduced by alloxan induced diabetes mellitus. However, SCEE diminished the toxic effects of diabetes and increased HDL level dose dependently with no significant difference in comparison to that of the normal control group at high dose.

Low density lipoprotein is considered toxic to the body and was significantly raised in comparison to that of the control group. SCEE dose dependently ameliorated the high level of LDL with no significant difference in comparison to the normal control group.

#### 3.4.4. Protective Role of SCEE against Alloxan Induced Toxicity of Liver

Alloxan is reported to cause hepatic toxicity besides inducing diabetes mellitus. SCEE was tested to ameliorate hepatic toxicity induced by alloxan in addition to diabetes correction. There was a significant increase in ALT, AST, and ALP level in alloxan induced diabetic rats in comparison to that of the normal control rats (Table 6). SCEE dose dependently reduced its serum level. At 300 mg/kg b.w. dose, a nonsignificant difference was observed when compared to glibenclamide treated group.

Similarly, there was a significant rise in level of LDH, bilirubin, and *γ*-GT in diabetic animals in comparison to that of the normal group (Table 7). Bilirubin level was observed to be significantly lower than diabetic group at high dose. In the case of *γ*-GT, its level was decreased but was significantly higher than normal control group. No significant difference was observed in comparison to the reference group at high dose of SCEE.

The effect of alloxan treatment on liver tissue protein, TBARS, H_2_O_2_, and nitrite content is shown in Table 8. The protein level was significantly decreased in diabetic animal group. It was restored by SCEE administration with a nonsignificant difference at high dose of 300 mg/kg b.w. in comparison to that of the normal control group. Similarly, TBARS content was markedly elevated in diabetic group. SCEE treatment notably diminished its content with no significant difference in comparison to glibenclamide treated group, but it was significantly higher when compared to that of the normal control group. Higher level of H_2_O_2_ and nitrite content was diminished by SCEE treatment and no significant difference was recorded in comparison to normal control group at high dose.

Diabetes mellitus significantly reduced antioxidant enzymes and GSH level (Table 9). CAT, POD, GST, GR, GP_X_, and GSH levels were increased significantly by SCEE at high dose but were significantly lower than the normal control group. However, SCEE high dose treated group showed no significant difference in comparison to glibenclamide treated group. The SOD was restored by SCEE completely at high dose.

**Figure 1:**
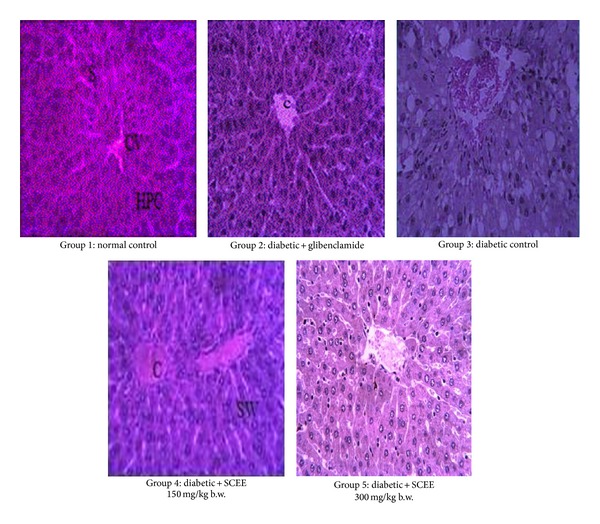
Microphotographs of liver histology of different groups after treatments of SCEE (H&E staining, 40x). CV: central venule, HPC: hepatocytes, S: sinosides, DLS: degeneration of lobular shape, FC: fatty change, CI: inflammatory cells infiltration, C: congestion, SW: sinusoids widening, and SCEE:* S. cordata* ethyl acetate fraction.

Alloxan induced toxicity in liver was observed at the morphological level by performing histology with H&E stain. Effect of SCEE on hepatic histomorphology is given in Figure 1. Alloxan induced alterations in histoarchitecture of liver. Congestion was noted in central venules of hepatic tissue. Steatosis was observed around the central venules with widening of sinusoids and inflammatory cells infiltration. Congestion was observed in central venules of the SCEE treated low dose treated group with mild inflammatory cells infiltrations and widening of sinosides but no steatosis. High dose SCEE treatment group expressed only mild congestion in central venule.

#### 3.4.5. Protective Role of SCEE against Alloxan Induced Toxicity of Pancreas

The protective role of SCEE against alloxan induced toxicity was assessed by stress markers, antioxidant enzymes, and histology. The effect of alloxan treatment on pancreas tissue protein, TBARS, H_2_O_2_, and nitrite content is displayed in Table 8. The protein level of pancreas tissue was significantly decreased after alloxan induction of diabetes but significantly increased by SCEE treatment. SCEE at high dose showed protein level with no significant difference to glibenclamide treated group but it was significantly lower than normal control.

TBARS, nitrite, and H_2_O_2_ were markedly higher in diabetic group. SCEE treatment notably diminished the level of these parameters and no significant difference was observed in comparison to glibenclamide treated group but it was significantly higher than normal control group. No significant decrease was noted in case of nitrite in SCEE treated groups.

Alloxan toxicity significantly reduced antioxidant enzymes and GSH level (Table 9). CAT, POD, SOD, GST, GP_X_, and GR levels were significantly increased by SCEE at high dose but were significantly lower than normal control group. However, no significant difference was observed in comparison to glibenclamide treated group. GSH exhibited complete recovery at high dose of SCEE.

**Figure 2:**
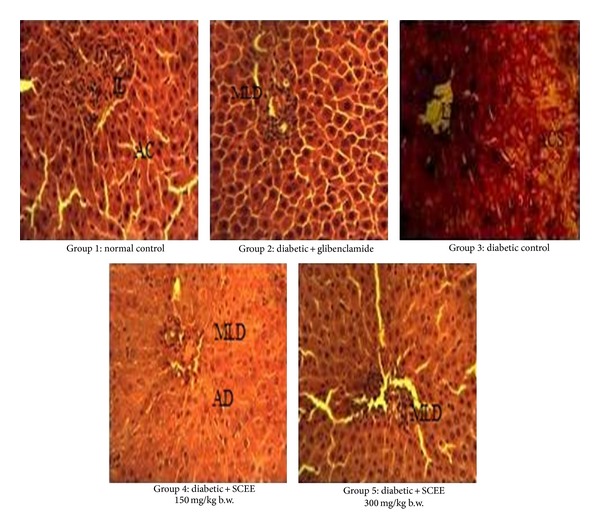
Microphotographs of pancreas histology of different groups after treatments of SCEE (H&E staining, 40x). IL: islet of Langerhans, AC: acinar cells, MLD: mild Langerhans disruption, AD: acinar disintegration, LD: Langerhans disruption, ACS: acinar cells steatosis, IC: inflammatory cells, and SCEE:* S. cordata* ethyl acetate fraction.

Normal control group pancreas illustrated in Figure 2 expresses compact islets of Langerhans, surrounded by acinar cells with prominent nuclei of the pancreas. Inflammatory cells infiltration was not observed. Alloxan administration resulted in the disruption of the islets of Langerhans and steatosis was observed in acinar cells. Inflammatory cell infiltration was seen in the acinar cells as well as islets of Langerhans. SCEE treatment showed a significant protection of islets of Langerhans and acinar cells in comparison to that of the diabetic group. Only mild disruptions were observed at high dose of SCEE while acinar cells were in normal morphological form.

#### 3.4.6. Protective Role of SCEE against Alloxan Induced Testicular Toxicity

The effect of alloxan treatment on testes tissue protein, TBARS, H_2_O_2_, and nitrite content is displayed in Table 8. The protein level of testes tissues was significantly low in diabetic group, while TBARS, H_2_O_2_, and nitrite contents were significantly higher than the normal control group. Protein level was significantly increased by SCEE treatment in a dose dependent manner. SCEE at high dose restored protein level with a nonsignificant difference in comparison to the control group. H_2_O_2_ and TBARS profile was also ameliorated by SCEE treatment in a dose dependent manner. Similarly, nitrite content was also significantly decreased after SCEE treatment but was significantly higher than the normal control group.

Antioxidant enzymes and GSH levels of different groups are displayed in Table 9. In result of oxidative stress, the level of GSH and antioxidant enzymes, that is, CAT, POD, SOD, GST, GP_X_, and GR, was significantly decreased. CAT, GSH, POD, and SOD profiles were completely restored by SCEE high dose. However, GST and GR enzymes profiles were equal to glibenclamide treated group but significantly different from normal control group. SCEE also expressed dose dependent improvement in GP_X_ level but neither equal to the normal control group nor glibenclamide treated group.

**Figure 3:**
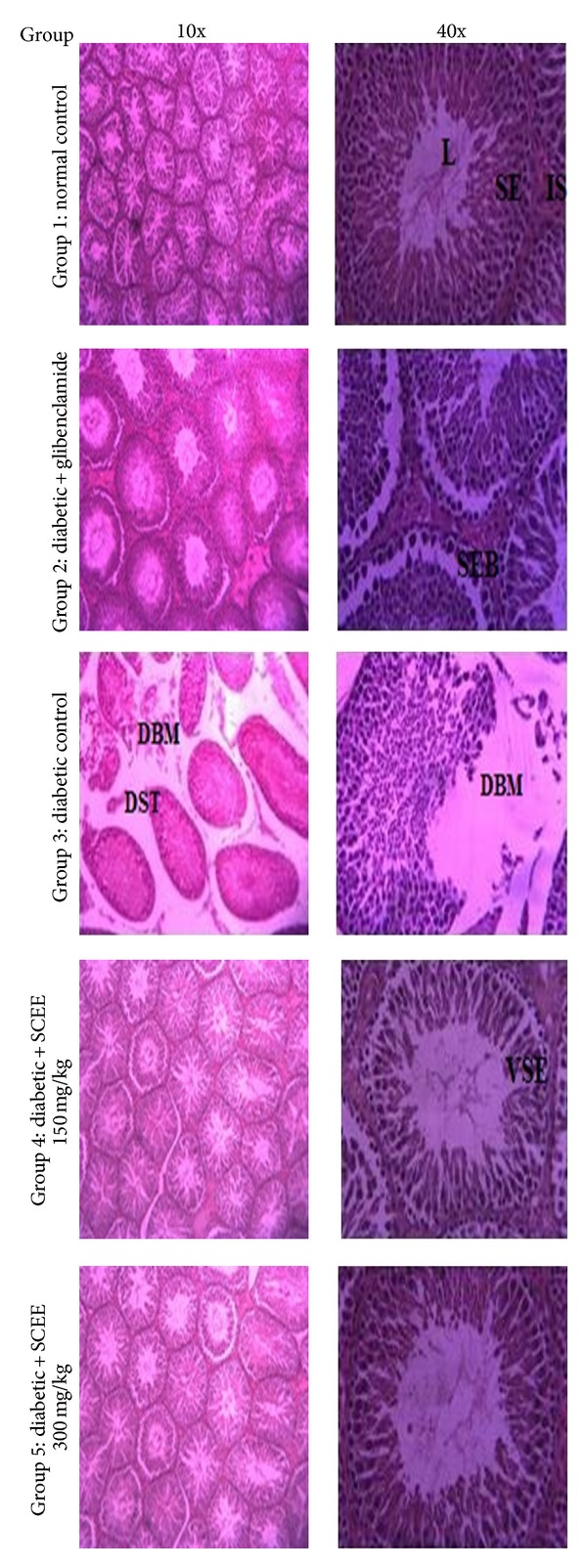
Microphotographs of testes histology of different groups after treatments of SCEE (H&E staining, 10x and 40x). L: lumen, IS: interstitium, SE: seminiferous epithelium, VSE: vacuolated seminiferous epithelium, DBM: disrupted basement membrane, SEB: seminiferous epithelium and basement membrane space, DST: displaced seminiferous tubules, and SCEE:* S. cordata* ethyl acetate fraction.

Effect of SCEE on testes histology in different groups is illustrated in Figure 3. Compactness of the seminiferous tubules was observed in control group. However, alloxan induced displacement and size and shape alterations of the seminiferous tubules. Disruptions of the seminiferous tubules were observed in the diabetic group. Leydig cells were also noted to be disrupted and in disorganized form. SCEE treatment showed a significant ameliorative role in terms of pathological alteration. Mild spaces between basement membrane and seminiferous epithelium were observed in SCEE treated groups. Germ cells density in seminiferous epithelium section was also observed in parallel state.

**Figure 4:**
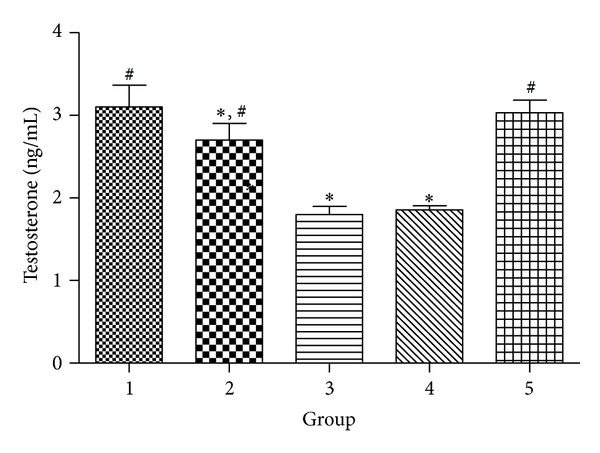
Effect of different treatments of SCEE on testosterone level in alloxan induced diabetic animals. 1: normal control, 2: diabetic + glibenclamide, 3: diabetic control, 4: diabetic + SCEE 150 mg/kg b.w., and 5: diabetic + SCEE 300 mg/kg b.w. ∗ and # indicate significant difference (*P* < 0.05) of different groups in comparison to normal control group and diabetic control group, respectively.

Effects of SCEE on testosterone level in different groups are illustrated in Figure 4. Alloxan treatment significantly (*P* < 0.05) reduced testosterone concentration in diabetic group compared with the normal control group. SCEE high dose treatment recovered testosterone level and a nonsignificant change was observed in comparison to the normal control group. However, glibenclamide treated group showed significantly low (*P* < 0.05) concentration in comparison to the normal control group.

## 4. Discussion

Diabetes is a major health issue influencing major population worldwide. In order to determine, whether SCEE has any role in diabetes mellitus, was evaluated by anti-hyperglcemic, hypoglycemic and chronic multiple dose experiments in rats. Antihyperglycemic and hypoglycemic activities were determined by blood glucose measuring at different intervals, while in chronic antidiabetic activity, besides glucose measuring various biochemical parameters and different tissue protection, analysis against alloxan induced toxicity was performed.

Epidemiological studies and clinical trials positively uphold the idea that hyperglycemia is the central cause for complications. Adequate blood glucose control is the key for averting or switching diabetic complexities and enhancing personal satisfaction in patients with diabetes. Consequently sustained decrease in hyperglycemia will diminish the danger of advancing microvascular difficulties and doubtlessly decrease the danger of macrovascular deforms [28]. On the basis of this articulation, we have chosen the glucose-prompted hyperglycemic model to screen the antihyperglycemic activity of the plants utilizing SCEE fraction of* S. cordata*. Any medicine that is successful in diabetes to control the ascent in glucose level by diverse mechanisms and the capacity of the fraction to avoid hyperglycemia could be investigated by glucose-loaded hyperglycemias model.

In the glucose-loaded hyperglycemias model, SCEE displayed significant antihyperglycemic action. The excessive amount of glucose in the blood impels insulin secretion. This secreted insulin will stimulate peripheral glucose utilization and controls the processing of glucose through numerous mechanisms [29]. However, from the study (glucose control) it was clear that the secreted insulin requires 2-3 hours to carry the glucose level to normal. On account of utilizing SCEE and glibenclamide treated animals, the glucose levels have not surpassed more those of the negative control group, giving an implication with respect to the strong activity of the SCEE and standard drug glibenclamide in glucose usage. The impact of glibenclamide on glucose tolerance has been credited to upgrade activity of *β*-cells of the pancreas for higher release of insulin. So the system behind the SCEE antihyperglycemic activity includes an insulin-like impact, likely, through high glucose utilization or upgrading the sensitivity of *β*-cells to glucose, secreting the elevated insulin amount [28]. Various plants with similar hypoglycemic activity have been reported [30].

Alloxan prompts diabetes by destroying the insulin secreting cells of the pancreas resulting in hypoinsulinemia and hyperglycemia [31]. Alloxan induces hyperglycemia by specific cytotoxic impact on pancreatic beta cells. One of the intracellular phenomena for its cytotoxicity is through the production of free radicals exhibited both* in vivo* and* in vitro* [32]. Our examinations demonstrate that the proficiency of the SCEE in the support of blood glucose levels in alloxan-instigated diabetic rats may be potentially by the aforementioned routes.

In uncontrolled or inadequately regulated diabetes, there is an elevated glycosylation of various proteins including haemoglobin. Glycosylated haemoglobin level is elevated in 16% of diabetes mellitus patients and the measure of increment was reported to be directly correlated with the fasting blood glucose level. In diabetes, the overabundant glucose in the blood reacts with haemoglobin. Consequently, the total haemoglobin level is diminished in alloxan diabetic rats [33]. Administration of SCEE for 15 days inhibited glycosylated haemoglobin and increased the level of total haemoglobin in diabetic rats. This could be because of the aftereffect of enhanced glycemic control processed by SCEE constituents.

The profile of serum lipids is often increased in diabetes mellitus and such an increase in lipids prompts coronary illness. This elevated level of serum lipids is because of the uninhibited activities of lipolytic hormones on the fat stores primarily because of the low activity of insulin. Under typical conditions, insulin endorses the catalyst lipoprotein lipase, which hydrolyzes triglycerides. Further, in diabetic state lipoprotein lipase is not initiated because of insulin inadequacy, stimulating hypertriglyceridemia [34]. Additionally, insulin lack is connected with hypercholesterolemia. Lack of insulin may account for dyslipidemia, since insulin has an inhibitory activity on HMG-CoA reductase, a key rate-constraining catalyst responsible for the metabolism of cholesterol-rich LDL particles. The systems accountable for the elevated hypertriglyceridemia and hypercholesterolemia in uncontrolled diabetes patients are because various metabolic aberrances happen sequentially [35]. In our study, diabetic rats indicated hypercholesterolemia and hypertriglyceridemia and the treatment with SCEE markedly diminished both cholesterol and triglyceride levels. This shows that SCEE treatment can counteract or be accommodating in decreasing the problems of lipid profile. These observations additionally back the theory that the action of SCEE may be directly involved in changes in insulin levels upon treatment [36].

The alloxan impelled hyperglycemia induces a rise of plasma levels of urea and creatinine contents, which are recognized as fundamental markers of renal dysfunction [37]. Our results also exhibited a noteworthy increase in the plasma urea and creatinine profiles in the diabetic group contrasted with that of the control group. These effects showed that diabetes may expedite renal malfunctioning. However, with administration of diabetic rats with SCEE, the levels of urea and creatinine were markedly decreased in comparison to the diabetic groups. This further affirms the utility of SCEE in diabetes-cohorted complications. Renal histology demonstrated tubular, corpuscular, and interstitial alterations. Alterations in the capsules were identified with the diminishment of Bowman's space which could be because of the enlargement of mesangial or endothelial cells of the glomerulus. Extension of mesangial spaces was portrayed by Hamada and Fukagawa [38] and Teoh et al. [39]. Prabhakar et al. [40] also reported extensive mesangial progression and thickening of basement membrane. The function of the mesangium is to back and stay in the capillary loop to empower them to hold their structure and capacity. Observations of Fioretto and Mauer [41] affirm that mesangial extension is pivotal structural change, prompting a malfunctioning of renal capacity in diabetes. It is suspected that such pathological developments decrease the capillary areas accessible for filtration [42]. Expansion in mesangium was indicated to associate with the advancement of proteinuria, despite the fact that Wolf et al. [42] were not certain that mesangial development is the main explanation for the proteinuria as they contend that the progressions additionally happen inside the visceral layer of Bowman's capsule which can accelerate changes in the glomerular filtration boundary. It is well understood that alterations in the structure and capacity of the glomerulus influence the tubules. Our observations illustrate more changes in the renal tubules like epithelial straightening vacuolization and dilatation. Comparative to our observations, Prabhakar et al. [40] reported tubular dilation and atrophy, Teoh et al. [39] reported hypercellularity and necrosis of the proximal tubules, and Baehr [43] and Saundby [44] portrayed epithelial vacuolization of the proximal tubules and the loop of Henle.

All these tubular alterations indicate an unsettling influence in their functional capacity. Our observations showed that treatment with SCEE can ameliorate the alterations induced by alloxan induced diabetes.

The increment in the profile of plasma AST, ALT, LDH, ALP, and *γ*-GT demonstrates that diabetes may induce hepatic malfunctioning. Supporting our finding, it has been observed by Larcan et al. [45] that liver was necrotized in diabetic patients. In this manner, the increase of AST, ALT, LDH, ALP, and *γ*-GT level in plasma may be fundamentally because of the spillage of these enzymes from the liver cytosol into the blood stream [46], which gives an implication on the hepatotoxic impact of alloxan. Nevertheless, SCEE treatment to diabetic rats initiated diminishment in the profiles of these markers in plasma. These findings are in concurrence with those observed by Ohaeri [47] in rats. Besides, the amelioration of the liver damage by oral treatment of SCEE could be affirmed through investigating their impact on the level of plasma bilirubin. The finding showed that the experimentally induced diabetes markedly (*P* < 0.05) elevated the level of plasma bilirubin. However, SCEE treatment induced a huge (*P* < 0.05) decrease in plasma bilirubin. Rana et al. [48] reported that the increment in plasma bilirubin may occur because of the abatement of liver uptake, conjugation, or increment of bilirubin formation.

As one of numerous chronic sicknesses, diabetes is broadly accepted to build oxidative stress. In diabetes, an expansion in oxidative stress arises because of the compromise of antioxidant enzymes and an increment in oxygen free radical preparation [49]. The instigation in the levels of free radicals in alloxan-diabetic rats and the abatement in these levels after administration of alloxan-diabetic rats with SCEE are in concurrence with the findings by Baynes and Thorpe [49], Kumari and Augusti [50], Sheweita et al. [51], Anwar and Meki [52], and Campos et al. [53]. Moreover, it was noted that a single treatment of alloxan processed a reduction in the action of the liver (SOD, GSR, GP_X_, GST, and GR) throughout the advancement of alloxan induced diabetes mellitus [54].

Liver antioxidant enzymes, that is, CAT, POD, SOD, GR, and GST, and GSH were recuperated with SCEE treatment in the liver tissue. The augmentation in the action of GST is dependable with the actuation in the production of free radicals. Elevated GST activity could be one of the guard systems in human beings to detoxify or kill the poisonous metabolites, for example, ketones forms, produced in the liver by the diabetes. Anwar and Meki [52] proposed that garlic oil might adequately normalize the impeded antioxidant enzymes status in streptozotocin prompted diabetes. The impacts of these antioxidant enzymes may be functional in deferring the deleterious impacts of diabetes as retinopathy, nephropathy, and neuropathy because of irregularity between free radicals and antioxidant enzymes frameworks. From the above outcomes, it could be presumed that SCEE fraction has the ability to normalize the blood glucose levels. Histopathological findings of the liver segments of alloxan-instigated diabetic rats demonstrated numerous pathological signs including cell vacuolization, fatty deposition, and lymphocyte invasions. Ragavan and Krishnakumari [55] observed periportal vacuolization with central putrefaction in the rodent liver treated with single intraperitoneal infusions of alloxan at a dose of 120 mg/kg b.w., while Khalil et al. [56] with a higher dose of 150 mg/kg b.w. noted complications of the hepatic lines and vacuolized hepatocytes with picnotic nuclei. Vinagre et al. [57] reported swollen hepatocytes with vacuolar cytoplasm and hypertrophic nuclei, sinusoidal dilatation, and lymphocyte invasions in the periportal areas of streptozotocin-actuated diabetic rodent (70 mg/kg b.w.). Similarly, Al-Rawi [58] showed sinusoidal dilatation, swollen hepatocytes with cytoplasmic vacuole, lymphocyte invasions around the gateway veins, and intense fibrosis with an easier measurement of streptozotocin (50 mg/kg b.w.).

Our findings demonstrated that the treatment of diabetic rats with SCEE produced a low number of vacuolized cells and level of vacuolization. These findings suggest a role of the SCEE in ameliorating toxicity of alloxan.

The present information expresses that alloxan treatment induced high oxidative effect as proved by the huge build in testicular lipid peroxidation and also a critical decline in testicular GSH and antioxidant enzymes including SOD, CAT, POD, GST, GP_X_, and GR contents. This reflects an inhibitory activity of alloxan on enzymes in the testes. Yadav et al. [32] reported that alloxan prompted decrease of SOD and CAT activity and increase of LPO in rodent erythrocytes. The decrease of testicular SOD activity could be traced to hyperglycemia as reported by Sharpe et al. [59] who observed that glucose impelled oxidative stress in numerous tissues is because of lack of enzymes cofactors, to be specific copper and zinc. Glutathione is vital in the regulation of the cell redox state and a decrease in its cellular level in diabetes has been attributed to be characteristic of high oxidative anxiety [60]. The reduction in testicular GSH levels in alloxan-treated rats could be to a limited extent, ascribed to the decrease of GR activity which is accountable for recovery of GSH from its oxidized structure. Blum and Fridovich [61] indicated that GR is inactivated by superoxide anion. In this way, testes holding lessened SOD activity may have elevated flux of superoxide radicals that possibly could harm GR and diminish GSH content in the testes. Furthermore, the abatement in GSH level may reflect an immediate response between GSH and free radicals created by alloxan. This is reliable with the GSH capacity to scavenge oxidants by binding them covalently.

Similarly, the diminished cell antioxidant status and the elevated testicular LPO, the fundamental deteriorating process in cells, could be the essential competitors for diminishing testosterone synthesis and its low level in plasma of alloxan-treated rats. This is in agreement with the observations that a diminished testicular nonantioxidant enzyme ascorbic acid in diabetes could be identified with low testosterone concentration [62]. The stress induced by the diabetic condition may actuate a decrease of gonadotropins, and as an indirect outcome of low level of gonadotropins, a low level of testosterone was noted. Babichev et al. [63] reported that deviation to the reproductive system in laboratory animals with diabetes is known to be cohorted with toxicity in the gonads, as well as with malfunctioning of the hypothalamo-hypophyseal axis. Consequently, a decrease in the antioxidant enzymes framework in alloxan-treated rats could be partnered with progressions in the testes as well as malfunctioning with the hypothalamo-hypophyseal axis.

It has been recommended that the thiol groups are essential in the intracellular and membrane redox state of the secretory capacity of endocrine tissue [64]. The present study demonstrated that SCEE maintained GSH substances and secured GR and accordingly upholds testicular functional capacities. Moreover, treatment of SCEE may launch regeneration of the nonenzymatic antioxidant components in the testes, which was finished by synthesis of GSH from its oxidized structure [65]. Besides, treatments of SCEE to alloxan-treated rats for 15 days gave typical levels of SOD. This is in parallel with findings of Zbrońska et al. [66]. The prophylactic impact of SCEE against alloxan-instigated reduction in testosterone is because of ameliorative oxidative stress in the testes. This is proved by the normal plasma testosterone level noted in alloxan cotreated SCEE rats. This is in agreement with Biswas et al. [62] who noted critical stimulation of steroid dehydrogenase action and an ascent in testosterone levels in rat testes treated with antioxidant ascorbic acid through regulating the testicular antioxidant status. Notwithstanding, the gonadotropin profile in alloxan-treated animal with SCEE fraction treatment needs assessment to illustrate particularly the system underlying steroidogenic reaction. This plausibility is under examination.

In conclusion, SCEE are workable in maintaining antioxidant enzymes status in testicular cells accelerating diminished oxidative status and cell damage started by the diabetes inducer alloxan through free radical processing. This is likely the case with human diabetes mellitus.

Alloxan impelled trial diabetes is a significant display for type 1 diabetes. It has been accounted for that diabetic problem showed in alloxan-affected animals are free radical intervened [31]. Diminished pancreatic antioxidant enzyme's action in diabetic animals was because of the explanation that pancreas being low in antioxidant enzymes in essence is helpless to ROS assault. Since antioxidant enzymes are low, alloxan intervened ROS resulted due to diminished antioxidant enzymes action. Administration of SCEE fraction restored antioxidant enzymes to standard level in the pancreas.

The hypoglycemic impact of SCEE fraction may be because of the presence of insulin-like substances in fractions [67] stimulating beta cells to generate more insulin [68] and abnormal amount of fibers which meddles with carbohydrate absorption [69] or the regenerative impact of SCEE on pancreatic tissue [70, 71]. H&E staining has been utilized to illuminate the impact of SCEE fraction on the pancreas. Histopathological investigation of diabetic rats indicated almost destruction of islets of Langerhans, which was because of the adequate dose of alloxan used in the study. Significant difference was observed in the islet of Langerhans of the diabetic untreated group and diabetic treated groups, which gives a possibility of regenerative action of SCEE on the islet of Langerhans. Further work is in progress to isolate active component from SCEE.

## Figures and Tables

**Table 1:** Antihyperglycemic activity of SCEE in glucose loaded normal animals.

Treatment	Dose (mg/kg)	Blood glucose concentration (mg/dL)			
		0 min	60 min	120 min	180 min
SCEE	150	84.3 ± 4.4	121.7 ± 2.8∗	115.0 ± 5.0∗	76.0 ± 5.2∗
	300	85.5 ± 5.0	103.3 ± 5.7∗	92.0 ± 7.0∗	56.7 ± 12.5∗
Normal animals	—	84.6 ± 5.2	170.4 ± 6.4	141.4 ± 3.6	112.5 ± 4.8

Values expressed as means ± SD. ∗Significance difference (*P* < 0.05) is expressed against normal control. SCEE: *S. cordata* ethyl acetate fraction.

**Table 2:** Antihyperglycemic activity of SCEE in glucose loaded alloxan induced diabetic animals.

Treatment	Dose (mg/kg)	Blood glucose concentration (mg/dL)			
		0 min	60 min	120 min	180 min
SCEE	150	252.0 ± 4.4	315.0 ± 5.0∗	321.7 ± 12.8∗	266.0 ± 5.2∗
	300	247.5 ± 5.0	292.0 ± 7.0∗	303.3 ± 5.7∗	236.7 ± 12.5∗
Nondiabetic	—	84.5 ± 4.5	140.0 ± 6.4	112.0 ± 3.6	92.0 ± 4.8
Diabetic control	—	240.0 ± 4.5	379.7 ± 9.5	410.0 ± 5.0	342.0 ± 19.6
Glibenclamide	5	233.0 ± 5.7	270.0 ± 6.2∗	295.0 ± 5.0∗	228.7 ± 3.2∗

Values expressed as means ± SD. ∗Significance difference (*P* < 0.05) against diabetic control. SCEE: *S. cordata* ethyl acetate fraction.

**Table 3:** Hypoglycemic activity of SCEE in normal fasting animals.

Treatment	Dose (mg/kg)	Blood glucose concentration (mg/dL)			
		0 min	60 min	120 min	180 min
SCEE	150	84.0 ± 1.5	67.5 ± 1.5∗	61 ± 1.5∗	59 ± 1.5∗
	300	82.2 ± 2.5	61.6 ± 1.5∗	51 ± 1.5∗	55 ± 1.7∗
Nondiabetic	—	81.6 ± 2.1	79.4 ± 1.3	77 ± 1.2	75 ± 1.0
Glibenclamide	5	84.5 ± 1.5	52.5 ± 2.5∗	47 ± 2.5∗	49 ± 1.7∗

Values expressed as means ± SD. ∗Significance difference (*P* < 0.05) is expressed against nondiabetic. SCEE: *S. cordata* ethyl acetate fraction.

**Table 4:** Effect of SCEE on insulin, glucose, haemoglobin, and glycosylated hemoglobin level in alloxan induced diabetic animal.

Group	Glucose (mg/dL)	Insulin (U/L)	Haemoglobin (g/dL)	Gly. haemoglobin (%)
Normal control	83.6 ± 4.0^d^	15.0 ± 1.0^a^	12.6 ± 0.6^a^	1.5 ± 0.1^f^
Diabetic + glibenclamide	92.0 ± 2.0^cd^	13.1 ± 1.0^ab^	10.3 ± 0.5^b^	1.8 ± 0.1^ef^
Diabetic control	241.6 ± 7.6^a^	6.0 ± 1.0^e^	5.8 ± 0.3^c^	4.3 ± 0.4^a^
Diabetic + SCEE 150 mg/kg	129.3 ± 5.3^b^	10.0 ± 1.0^cd^	10.6 ± 0.5^ab^	3.1 ± 0.1^c^
Diabetic + SCEE 300 mg/kg	95.6 ± 3.5^cd^	12.5 ± 0.5^b^	12.0 ± 1.0^ab^	2.1 ± 0.1^e^

Values expressed as means ± SD. Means ± SD with different superscript letters (a–d) within the column indicate significant difference (*P* < 0.05). SCEE: *S. cordata* ethyl acetate fraction.

**Table 5:** Effect of SCEE on lipid level in alloxan induced diabetic animal.

Group	Triglycerides (mg/dL)	Total cholesterol (mg/dL)	HDL (mg/dL)	LDL (mg/dL)
Normal control	93.6 ± 4.1^d^	124.6 ± 4.7^e^	74.3 ± 5.1^a^	31.6 ± 6.6^d^
Diabetic + glibenclamide	109.3 ± 4.0^c^	135.6 ± 4.0^de^	67.6 ± 2.5^a^	46.1 ± 1.2^cd^
Diabetic control	201.6 ± 7.6^a^	228.3 ± 10.4^a^	40.6 ± 4.0^c^	150.6 ± 13.4^a^
Diabetic + SCEE 150 mg/kg	141.0 ± 3.6^b^	165.6 ± 4.0^bc^	55.6 ± 4.2^b^	81.8 ± 1.5^b^
Diabetic + SCEE 300 mg/kg	115.0 ± 3.8^c^	142.6 ± 2.5^d^	69.0 ± 3.6^a^	38.8 ± 1.2^d^

Values expressed as means ± SD. Means ± SD with different superscript letters (a–d) within the column indicate significant difference (*P* < 0.05). SCEE: *S. cordata* ethyl acetate fraction.

**Table 6:** Effect of SCEE on liver serum markers level in alloxan induced diabetic animal.

Treatment	ALT (U/L)	AST (U/L)	ALP (U/L)
Normal control	37.5 ± 2.0^d^	42.6 ± 2.5^d^	57.3 ± 2.5^e^
Diabetic + glibenclamide	40.3 ± 1.5^cd^	51.0 ± 1.0^cd^	66.0 ± 3.6^de^
Diabetic control	85.0 ± 5.0^a^	105.0 ± 5.0^a^	112.0 ± 2.6^a^
Diabetic + SCEE 150 mg/kg	65.0 ± 5.0^b^	84.3 ± 5.1^b^	78.6 ± 5.1^bc^
Diabetic + SCEE 300 mg/kg	42.6 ± 2.5^cd^	55.0 ± 5.0^c^	62.3 ± 2.5^de^

Values expressed as means ± SD. Means ± SD with different superscript (a–e) letters within the column indicate significant difference (*P* < 0.05). SCEE: *S. cordata* ethyl acetate fraction.

**Table 7:** Effect of SCEE on LDH, bilirubin, and *γ*-GT level in alloxan induced diabetic animal.

Treatment	LDH (U/L)	Bilirubin (mg/mL)	*γ*-GT
Normal control	228.3 ± 7.6^f^	0.35 ± 0.05^d^	1.8 ± 0.1^d^
Diabetic + glibenclamide	248.3 ± 7.6^ef^	0.55 ± 0.05^cd^	2.2 ± 0.1^c^
Diabetic control	360.0 ± 15.0^a^	1.50 ± 0.10^a^	3.8 ± 0.1^a^
Diabetic + SCEE 150 mg/kg	293.3 ± 7.6^bc^	0.66 ± 0.05^c^	2.9 ± 0.1^b^
Diabetic + SCEE 300 mg/kg	264.3 ± 5.1^de^	0.40 ± 0.10^d^	2.5 ± 0.1^c^

Values expressed as means ± SD. Means ± SD with different superscript letters (a–f) within the column indicate significant difference (*P* < 0.05). SCEE: *S. cordata* ethyl acetate fraction.

**Table 8:** Effect of SCEE on pancreas, liver, and testis tissue protein, TBARS, H_2_O_2_, and nitrite content in alloxan induced diabetic animals.

Group	Protein	TBARS	H_2_O_2 _	Nitrite content
	(*μ*g/mg tissue)	(nM/min/mg protein)	(nM/min/mg tissue)	(*μ*M/mL)
Pancreas				
Normal control	5.0 ± 0.5^a^	1.9 ± 0.2^d^	5.5 ± 0.5^d^	64.6 ± 2.0^e^
Diabetic + glibenclamide	4.1 ± 0.3^bc^	2.3 ± 0.2^cd^	6.9 ± 0.4^cd^	70.3 ± 0.6^de^
Diabetic control	1.9 ± 0.2^e^	4.9 ± 0.4^a^	12.5 ± 0.5^a^	102.3 ± 2.5^a^
Diabetic + SCEE 150 mg/kg	2.6 ± 0.3^de^	3.5 ± 0.2^b^	9.7 ± 0.7^b^	91.3 ± 3.0^b^
Diabetic + SCEE 300 mg/kg	4.2 ± 0.3^b^	2.6 ± 0.2^c^	6.8 ± 0.3^cd^	74.0 ± 1.7^cd^

Liver				
Normal control	6.6 ± 0.5^a^	2.4 ± 0.1^e^	6.5 ± 0.4^e^	56.8 ± 1.9^d^
Diabetic + glibenclamide	5.2 ± 0.4^b^	2.9 ± 0.1^d^	8.3 ± 0.7^cd^	62.0 ± 1.8^cd^
Diabetic control	2.2 ± 0.3^d^	6.9 ± 0.3^a^	12.9 ± 0.9^a^	97.0 ± 3.6^a^
Diabetic + SCEE 150 mg/kg	3.5 ± 0.4^c^	4.7 ± 0.3^c^	9.4 ± 0.5^bc^	77.6 ± 2.5^b^
Diabetic + SCEE 300 mg/kg	5.7 ± 0.3^ab^	2.8 ± 0.1^de^	7.5 ± 0.5^de^	63.6 ± 0.6^cd^

Testis				
Normal control	4.7 ± 0.3^a^	1.5 ± 0.2^c^	5.3 ± 0.3^d^	57.5 ± 2.7^d^
Diabetic + glibenclamide	3.9 ± 0.2^b^	2.0 ± 0.1^c^	6.3 ± 0.6^cd^	64.6 ± 1.5^cd^
Diabetic control	1.7 ± 0.2^d^	5.6 ± 0.3^a^	11.4 ± 0.6^a^	93.3 ± 4.1^a^
Diabetic + SCEE 150 mg/kg	2.6 ± 0.4^c^	3.8 ± 0.2^b^	8.4 ± 0.5^b^	82.2 ± 4.0^b^
Diabetic + SCEE 300 mg/kg	4.2 ± 0.2^ab^	2.1 ± 0.6^c^	6.3 ± 0.3^cd^	66.0 ± 1.7^c^

Values expressed as means ± SD. Means ± SD with different superscript letters (a–d) within the column indicate significant difference (*P* < 0.05). SCEE: *S. cordata* ethyl acetate fraction.

**Table 9:** Effect of SCEE on pancreas, liver, and testis tissue antioxidant enzymes and GSH level in alloxan induced diabetic animals.

Group	CAT (U/min)	POD (U/min)	SOD (U/mg protein)	GST (nM/min/mg protein)	GSH (*μ*M/g tissue)	GPx (nM/min/mg protein)	GR (nM/min/mg protein)
Pancreas							
Normal control	7.8 ± 0.72^a^	6.5 ± 0.50^a^	6.8 ± 0.76^a^	118.3 ± 7.6^a^	36.7 ± 1.3^a^	83.3 ± 3.0^a^	154.0 ± 6.5^a^
Diabetic + glibenclamide	5.5 ± 0.43^b^	5.7 ± 0.30^ab^	5.2 ± 0.20^b^	104.0 ± 3.6^b^	35.9 ± 1.0^a^	75.6 ± 2.5^ab^	140.3 ± 5.5^ab^
Diabetic control	2.7 ± 0.21^d^	2.0 ± 0.25^d^	1.8 ± 0.15^e^	60.0 ± 5.0^d^	13.3 ± 1.5^d^	39.0 ± 2.6^e^	77.3 ± 2.5^d^
Diabetic + SCEE 150 mg/kg	3.7 ± 0.30^cd^	3.5 ± 0.41^c^	4.0 ± 0.11^cd^	80.0 ± 4.9^c^	25.0 ± 1.0^b^	46.0 ± 3.6^de^	99.3 ± 5.1^c^
Diabetic + SCEE 300 mg/kg	6.5 ± 0.42^b^	5.2 ± 0.25^b^	5.5 ± 0.40^b^	98.3 ± 2.5^b^	33.7 ± 1.1^a^	66.0 ± 3.8^c^	136.0 ± 3.6^b^

Liver							
Normal control	9.0 ± 0.20^a^	8.2 ± 0.37^a^	11.4 ± 0.85^a^	117.6 ± 5.2^a^	51.7 ± 3.4^a^	99.6 ± 4.5^a^	156.0 ± 6.5^a^
Diabetic + glibenclamide	7.7 ± 0.32^b^	7.2 ± 0.36^b^	9.6 ± 0.32^b^	93.6 ± 5.3^b^	49.4 ± 3.0^ab^	88.0 ± 3.0^ab^	137.3 ± 7.5^b^
Diabetic control	3.3 ± 0.32^d^	4.2 ± 0.45^d^	4.2 ± 0.50^d^	51.4 ± 8.0^d^	20.8 ± 1.6^e^	42.0 ± 2.0^d^	72.0 ± 6.2^d^
Diabetic + SCEE 150 mg/kg	5.2 ± 0.70^c^	5.6 ± 0.45^c^	6.8 ± 0.32^c^	69.9 ± 8.9^c^	34.2 ± 1.8^d^	57.6 ± 5.5^c^	99.0 ± 6.5^c^
Diabetic + SCEE 300 mg/kg	7.4 ± 0.41^b^	7.6 ± 0.32^ab^	10.1 ± 0.51^ab^	96.9 ± 5.8^b^	44.6 ± 2.3^bc^	79.0 ± 5.1^b^	133.3 ± 7.7^b^

Testis							
Normal control	8.6 ± 0.57^a^	6.9 ± 0.40^a^	8.7 ± 0.75^a^	127.6 ± 4.9^a^	44.0 ± 1.9^a^	75.0 ± 4.3^a^	162.6 ± 7.3^a^
Diabetic + glibenclamide	7.0 ± 0.6^b^	5.9 ± 0.17^ab^	6.8 ± 0.35^bc^	108.9 ± 3.9^b^	40.9 ± 2.4^ab^	65.3 ± 2.0^b^	145.0 ± 9.1^ab^
Diabetic control	3.3 ± 0.34^c^	2.6 ± 0.36^d^	2.9 ± 0.30^f^	61.8 ± 7.5^d^	17.3 ± 2.3^d^	31.3 ± 1.5^e^	76.0 ± 6.0^d^
Diabetic + SCEE 150 mg/kg	4.6 ± 0.55^c^	4.2 ± 0.70^c^	5.4 ± 0.26^de^	82.5 ± 5.4^c^	29.2 ± 2.4^c^	40.6 ± 2.0^d^	107.0 ± 4.3^c^
Diabetic + SCEE 300 mg/kg	7.3 ± 0.23^ab^	6.2 ± 0.21^ab^	7.6 ± 0.49^ab^	106.2 ± 5.7^b^	38.4 ± 2.1^ab^	58.0 ± 2.2^c^	141.3 ± 9.0^b^

Values expressed as means ± SD. Means ± SD with different superscript letters (a–e) within the column indicate significant difference (*P* < 0.05). SCEE: *S. cordata* ethyl acetate fraction.
